# Disadvantaged Background Status and the Experiences of Academic Anesthesiologists

**DOI:** 10.1001/jamanetworkopen.2026.4796

**Published:** 2026-04-02

**Authors:** Cody R. Sain, Earl F. Goldsborough, Megan F. Hunt, Sashini K. Godage, Morgan T. Sexton, Jingui A. He, Kelsey M. Flowers Zachos, Angela M. Bader, Kristin L. Schreiber, Morana Lasic

**Affiliations:** 1 Mass General Brigham, Brigham and Women’s Hospital, Department of Anesthesiology, Perioperative and Pain Medicine, Harvard Medical School, Boston, Massachusetts; 2 Department of Anesthesiology, Perioperative, and Pain Medicine, Stanford University School of Medicine, Stanford, California

## Abstract

This survey study examines disadvantaged background status and the experiences of workplace inclusion and harassment among academic anesthesiologists compared with their nondisadvantaged colleagues.

## Introduction

Little is known about the diversity of socioeconomic status (SES) among the US physician workforce, with no known studies exploring how socioeconomic background impacts the academic medicine experience within DEIB (diversity, equity, inclusion, and belonging) scholarship. SES of the physician workforce likely trends with US medical students, as the top 2 income quintiles account for most matriculants.^1^ Using the National Institutes of Health (NIH) disadvantaged background (DB) as a formal proxy for SES,^2^ this pilot study assesses the experiences of workplace inclusion and harassment among DB-identifying anesthesiologists compared with their colleagues.

## Methods

We conducted this survey study across a single anesthesiology department using an adapted questionnaire from a prior Stanford survey.^3^ Stanford’s 2021 DEI Survey was designed by Stanford’s Office of Institutional Research and Decision Support alongside DEI experts through a year-long process, including community feedback and review of existing survey research.^4^ Questionnaire modifications included additional demographics (including DB) and removing open-ended fields. The NIH defines a DB using criteria focused on economic need and life experiences, including a history of homelessness, receipt of federal assistance, limited parental education, and living in rural or physician-shortage areas.^2^ Development of the survey followed best practices outlined by the AAPOR reporting guideline, ensuring questions were free of bias, arranged in a logical order without attempting to influence responses, and ensuring the confidentiality of respondents.^5^ Participation implied consent. This study was deemed exempt by the Mass General Brigham institutional review board due to the voluntary and anonymous nature of the questionnaire.

Questionnaires were distributed via REDCap (eAppendix in Supplement 1) to 132 faculty members and 135 trainees in the Department of Anesthesiology at Brigham and Women’s Hospital. Email reminders were sent weekly from April 8 to May 20, 2024. Responses were anonymous to researchers. Respondents answered yes or no items and indicated agreement using a 5-point Likert scale (1-strongly disagree to 5-strongly agree). For binary items (ie, yes or no), asymptomatic 2-sided Pearson χ^2^ tests were used to evaluate differences. For ordinal items (ie, Likert), responses were averaged to generate mean (SD) scores, and differences were assessed using asymptomatic 2-sided Mann-Whitney U tests. Statistical analysis was completed using SPSS Statistics version 29.0.2.0 (20) (IBM). Statistical significance was set at *P* < .05. Data were analyzed from June to September 2024.

## Results

Of the 267 questionnaires distributed, 190 faculty or trainees (71.2%) responded, and 34 (17.9%) identified as coming from a DB (Table 1). DB status was associated with a greater sense of exclusion (55.2% vs 20.1%; *P* < .001), experiences of verbal harassment (41.9% vs 22.8%; *P* = .03), discrimination (20.7% vs 8.3%, *P* = .047), racial invalidation (33.3% vs 11.1%; *P* = .002), and microaggressions of being othered (45.2% vs 19.6%; *P* = .003) (Table 2). DB status was also associated with mean (SD) scores for feeling less valued (3.63 [1.16] vs 4.08 [1.04]; *P* = .02), more rejection for being different (2.81 [1.38] vs 2.28 [1.16]; *P* = .048), and a sense of working harder for equal evaluations (3.19 [1.45] vs 2.50 [1.25]; *P* = .01) and being recognized as academic (3.35 [1.31] vs 2.58 [1.09]; *P* = .001) compared with their non-DB colleagues (Table 2).

**Table 1.** Demographic Characteristics of Questionnaire Respondents

Characteristic	Total No. (%), (n = 190)
Role	
Faculty	82 (43.2)
Trainees (residents and fellows)	108 (56.8)
Disadvantaged background status	
Yes	34 (17.9)
No	146 (76.8)
Prefer not to say	10 (5.3)

The table presents the distribution of respondents by role (faculty or trainees) and disadvantaged background status.

**Table 2.** Workplace Experiences and Climate Between DB and Non–DB Respondents

Experiences	Participant, No. (%)		*P* value^c^
	DB^a^	Non-DB^b^	
Participants responding "yes" to binary survey items, No. (%)^d^			
Exclusion	16 (55.2)	29 (20.1)	<.001
Verbal harassment	13 (41.9)	33 (22.8)	.03
Physical harassment	2 (6.7)	2 (1.4)	.08
Discrimination	6 (20.7)	12 (8.3)	.047
Racial invalidation	10 (33.3)	16 (11.1)	.002
Inferior	10 (33.3)	27 (19.0)	.08
Othered	14 (45.2)	28 (19.6)	.003
Questionnaire items measured on 5-point Likert scale, mean (SD)^d^			
Feeling valued	3.63 (1.16)	4.08 (1.04)	.02
Psychological safety	3.53 (1.24)	3.98 (0.95)	.08
Mutual respect	3.94 (1.06)	4.25 (0.93)	.09
Rejection for difference	2.81 (1.38)	2.28 (1.16)	.048
Scrutiny	2.87 (1.43)	2.85 (1.32)	.95
Inequitable evaluation	3.19 (1.45)	2.50 (1.25)	.01
Inequitable perception	3.35 (1.31)	2.58 (1.09)	.001

Abbreviation: DB, disadvantaged background.

^a^ Sample sizes ranged from 29 to 32.

^b^ Sample sizes ranged from 142 to 145.

^c^ Statistical significance was set at *P* < .05.

^d^ The survey questions and responses assessing each experience can be found in the eAppendix in Supplement 1.

## Discussion

In this survey study, respondents with DB status faced significant workplace challenges compared with their counterparts. With the NIH definition, research on the DB group becomes feasible and is crucial for understanding its effects on the diversity of the US physician workforce. Economic background may intersect with other minority backgrounds often highlighted in diversity scholarship, including race, ethnicity, gender identity, and sexual orientation, resulting in intersectional identities uniquely vulnerable to adverse workplace climates.^6^ Limitations include the study of a single center and specialty, nonresponse bias, and limited sample size, impacting the ability to study confounding and intersectional variables. In this exploratory study, we aim to highlight that DB status may play an independent or additive role in the experiences of minority anesthesiologists. Future studies should focus on larger, more diverse samples across academic medicine and disaggregate data analysis by trainee and faculty status.

## Notes

Supplement 1. **eAppendix.** BWH Anesthesiology Culture and Climate Survey

Supplement 2.
Data Sharing Statement
