# Supplementary material for: Disadvantaged Background Status and the Experiences of Academic Anesthesiologists
Source: JAMA Netw Open. 2026 Apr 2;9(4):e264796. doi: 10.1001/jamanetworkopen.2026.4796 (PMC13047457; doi:10.1001/jamanetworkopen.2026.4796)
Supplement: Supplement 1. — eAppendix. BWH Anesthesiology Culture and Climate Survey [file jamanetwopen-e264796-s001.pdf]

Supplemental Online Content

Sain CR, Goldsborough EF, Hunt MF, et al. Disadvantaged background status and the experiences of academic anesthesiologists. *JAMA Netw Open.* 2026;9(4):e264796. doi:10.1001/jamanetworkopen.2026.4796

eAppendix. BWH Anesthesiology Culture and Climate Survey

This supplemental material has been provided by the authors to give readers additional information about their work.

© 2026 Sain C et al. *JAMA Network Open*.

BWH Anesthesiology Culture & Climate Survey

This anonymous and confidential DEIB (diversity, equity, inclusion, and belonging) Annual Culture & Climate Survey is about your experience working & training in our department.

Thank you for your time!

Morana Lasic & Cody Sain

REGARDING FORMAL REPORTING MECHANISMS

Please note, survey feedback is anonymous and confidential. While we encourage candid feedback, this survey should not be used as a reporting mechanism and no one from the university or hospital will be able to follow-up with you directly based on your responses. Survey responses will be anonymized and presented as themes.

We are committed to creating an inclusive workplace that is free of discrimination, bias and harassment where all individuals feel respected and safe. If you have a concern about your workplace experience, please let us know. Reporting options include notifying your supervisor, any manager, MGB Compliance or Human Resources via the 'Report a Concern' tile on Vitals.

Demographics Establishing Affiliation

I currently hold the position of

- ◯ Faculty
- ◯ Resident
- ◯ Fellow
- ◯ Researcher (non-clinicians)
- ◯ CRNA/SRNA
- ◯ Anesthesia Tech
- ◯ Administrative Staff
- ◯ Prefer not to say

How long have you been a member of the BWH Department of Anesthesiology?

- ◯ < 5 years
- ◯ 5-10 Years
- ◯ 10-20 Years
- ◯ 20+ years
- ◯ Prefer not to say

Demographics Race & Ethnicity, Origin, and Native Language

We would like to know more about your race, ethnic heritage, or national origin.

© 2026 Sain C et al. *JAMA Network Open*.

Which of the following racial or ethnic groups do you identify with?
(Mark all that apply)

- ☐ American Indian or Alaska Native (e.g., Navajo Nation, Blackfeet Tribe, Inupiat Traditional Gov't., etc.)
- ☐ Asian or Asian American (e.g., Chinese, Japanese, Filipino, Korean, South Asian, Vietnamese, etc.)
- ☐ Black or African American (e.g., Jamaican, Nigerian, Haitian, Ethiopian, etc.)
- ☐ Hispanic or Latine/x (e.g., Puerto Rican, Mexican, Cuban, Salvadoran, Colombian, etc.)
- ☐ Middle Eastern or North African (e.g., Lebanese, Iranian, Egyptian, Moroccan, Israeli, Palestinian, etc.)
- ☐ Native Hawaiian or Pacific Islander (e.g., Samoan, Guamanian, Chamorro, Tongan, etc.)
- ☐ White or European (e.g., German, Irish, English, Italian, Polish, French, etc.)
- ☐ Prefer not to say
- ☐ Other

My race or ethnicity is best described as

____

Is English your native language?

- ◯ Yes
- ◯ No
- ◯ Prefer not to say

Demographics Gender & Sexual Orientation

Which best describes your gender identity?
(Mark all that apply)

- ☐ Gender nonconforming
- ☐ Genderqueer
- ☐ Man
- ☐ Nonbinary
- ☐ Questioning
- ☐ Woman
- ☐ Prefer not to say
- ☐ Other

My gender identity is best described as

____

Do you identify as transgender or under the trans umbrella?

- ◯ Yes
- ◯ No
- ◯ Prefer not to say

Do you identify as a member of the LGBTQIA+ Community?

- ◯ Yes
- ◯ No
- ◯ Prefer not to say

Which best describes your sexual identity/orientation?

(Mark all that apply)

- ☐ Asexual
- ☐ Bisexual
- ☐ Gay
- ☐ Heterosexual or Straight
- ☐ Lesbian
- ☐ Pansexual
- ☐ Queer
- ☐ Questioning
- ☐ Other
- ☐ Prefer not to say

My sexual identity is best described as

____

 © 2026 Sain C et al. *JAMA Network Open*.

Demographics Education

What best describes your highest level of education?

- ◯ Less than high school
- ◯ High School
- ◯ 2 year college degree
- ◯ 4 year college degree
- ◯ Master's Degree
- ◯ Professional or doctoral degree
- ◯ Prefer not to say

What is the highest level of education achieved by any of your parents or guardians during your childhood (age 0-18)?

- ◯ Less than high school
- ◯ High School
- ◯ 2 year college degree
- ◯ 4 year college degree
- ◯ Master's Degree
- ◯ Professional or doctoral degree
- ◯ Not sure
- ◯ Prefer not to say

Demographics Socioeconomic Status & Disadvantaged Background

Do you identify as coming from a low-income or low socioeconomic status (SES) background?

- ◯ Yes
- ◯ No
- ◯ Prefer not to say

© 2026 Sain C et al. *JAMA Network Open*.

Do you identify as coming from a disadvantaged background as defined by the NIH (see below)?<br><br>Definition:<br>NIH encourages institutions to diversify their student and faculty populations to enhance the participation of individuals from groups that are underrepresented in the biomedical, clinical, behavioral and social sciences, including individuals from disadvantaged backgrounds. Individuals from disadvantaged backgrounds are defined as those who meet TWO or more of the following criteria:<br><br>1. Were or currently are homeless, as defined by the McKinney-Vento Homeless Assistance Act;<br><br>2. Were or currently are in the foster care system, as defined by the Administration for Children and Families;<br><br>3. Were eligible for the Federal Free and Reduced Lunch Program for two or more years;<br><br>4. Have/had no parents or legal guardians who completed a bachelor's degree;<br><br>5. Were or currently are eligible for Federal Pell grants;<br><br>6. Received support from the Special Supplemental Nutrition Program for Women, Infants and Children (WIC) as a parent or child;<br><br>7. Grew up in one of the following areas: a) a U.S. rural area, as designated by the Health Resources and Services Administration (HRSA) Rural Health Grants Eligibility Analyzer, or b) a Centers for Medicare and Medicaid Services-designated Low-Income and Health Professional Shortage Areas (qualifying zip codes are included in the file). Only one of the two possibilities in #7 can be used as a criterion for the disadvantaged background definition.	◯ Yes<br>◯ No<br>◯ Prefer not to say

Demographics Age, Parental Status, & Disability

Please indicate your current age bracket.	◯ 18-22 years old<br>◯ 23-29 years old<br>◯ 30-39 years old<br>◯ 40-49 years old<br>◯ 50-65 years old<br>◯ 65+ years old<br>◯ Prefer not to say
Do you identify as a Parent or Legal Guardian?	◯ Yes<br>◯ No<br>◯ Prefer not to say

© 2026 Sain C et al. *JAMA Network Open*.

Do you identify as a person with a disability?

- ◯ Yes
- ◯ No
- ◯ Prefer not to say

If you have a disability and are comfortable sharing, please rate how much you agree with the following statement.

"I feel that my disability is being adequately accommodated by our department."

- ◯ Strongly Disagree
- ◯ Somewhat Disagree
- ◯ Neither Agree nor Disagree
- ◯ Somewhat Agree
- ◯ Strongly Agree
- ◯ Prefer not to say

We would like to understand the financial pressure that our BWH community members typically face.

Financial Pressures

I or my family would not be able to afford an unexpected expense of ---------------- .

$0 $10,000 $20,000

(Place a mark on the scale above)

Do any of these statements describe a financial pressure you have faced during the past ONE year?

(Mark all that apply)

- ☐ I have faced financial pressures, but the financial pressures I faced are not listed below
- ☐ I would be financially burdened if I needed to send funds home to support my family or loved ones
- ☐ I have not been able to cover housing costs
- ☐ I have not been able to afford enough food
- ☐ I did not participate in an educational or work-related activity because the costs were too high
- ☐ I did not participate in a social activity because the costs were too high
- ☐ I have not been able to cover routine transportation costs
- ☐ I was not able to afford necessary medical care
- ☐ I was not able to cover funds to travel to see family or loved ones
- ☐ I do not face any financial pressures
- ☐ Prefer not to say

If comfortable, please specify the financial pressures you face below.

Welcome & Unwelcome Spaces

Note: If you select agree, there are follow up questions.

I have found one or more communities, groups, or spaces at BWH Department of Anesthesiology where I feel welcomed.

- ◯ Agree
- ◯ Disagree
- ◯ Prefer not to say

Please let us know the name(s) of communities, groups, or spaces at BWH Department of Anesthesiology where you feel most welcomed.

© 2026 Sain C et al. *JAMA Network Open*.

I have found one or more communities, groups, or spaces at BWH Department of Anesthesiology where I feel marginalized or excluded.

- ◯ Agree
- ◯ Disagree
- ◯ Prefer not to say

If you feel comfortable, please name the communities, groups, or spaces at BWH Department of Anesthesiology where you feel most marginalized or excluded.

Value & Psychological Safety

I feel valued as an individual at BWH Department of Anesthesiology.

- ◯ Strongly Disagree
- ◯ Somewhat Disagree
- ◯ Neither agree nor disagree
- ◯ Somewhat Agree
- ◯ Strongly Agree
- ◯ Prefer not to say

Please rate your agreement with the following statements:

In my department or work unit, it is usually easy to discuss difficult issues and problems.

- ◯ Strongly Disagree
- ◯ Somewhat Disagree
- ◯ Neither agree nor disagree
- ◯ Somewhat Agree
- ◯ Strongly Agree
- ◯ Prefer not to say

In my department or work unit, people are sometimes rejected for being different.

- ◯ Strongly Disagree
- ◯ Somewhat Disagree
- ◯ Neither agree nor disagree
- ◯ Somewhat Agree
- ◯ Strongly Agree
- ◯ Prefer not to say

Members of my department or work unit value and respect each other's contributions.

- ◯ Strongly Disagree
- ◯ Somewhat Disagree
- ◯ Neither agree nor disagree
- ◯ Somewhat Agree
- ◯ Strongly Agree
- ◯ Prefer not to say

I often feel under scrutiny by the people in my department or work unit.

- ◯ Strongly Disagree
- ◯ Somewhat Disagree
- ◯ Neither agree nor disagree
- ◯ Somewhat Agree
- ◯ Strongly Agree
- ◯ Prefer not to say

I feel as though I have to work harder than my colleagues to receive a comparable evaluation.

- ◯ Strongly Disagree
- ◯ Somewhat Disagree
- ◯ Neither agree nor disagree
- ◯ Somewhat Agree
- ◯ Strongly Agree
- ◯ Prefer not to say

© 2026 Sain C et al. *JAMA Network Open*.

I have to work harder than others to be perceived as academic in my department.

- ◯ Strongly Disagree
- ◯ Somewhat Disagree
- ◯ Neither agree nor disagree
- ◯ Somewhat Agree
- ◯ Strongly Agree
- ◯ Prefer not to say

Verbal Harassing Behavior

REGARDING FORMAL REPORTING MECHANISMS

Please note, survey feedback is anonymous and confidential. While we encourage candid feedback, this survey should not be used as a reporting mechanism and no one from the university or hospital will be able to follow-up with you directly based on your responses. Survey responses will be anonymized and presented as themes.

We are committed to creating an inclusive workplace that is free of discrimination, bias and harassment where all individuals feel respected and safe. If you have a concern about your workplace experience, please let us know. Reporting options include notifying your supervisor, any manager, MGB Compliance or Human Resources via the 'Report a Concern' tile on Vitals.

The next section will ask you to reflect on the last year.

We know it can be difficult to know for sure whether or not someone's behaviors toward you are attributable to any one of your identities or attributes. For some, it is very clear; for others, there is more uncertainty. We also know that this uncertainty can make the experiences even more difficult.

During the last year, have you ever experienced any verbal, written, or online harassing behaviors by someone associated with your BWH Workplace?

Note: Respondents who selected "Yes" will be asked some follow up questions.

Verbal, written, or online harassing behaviors examples:

1. Derogatory remark or gesture in person or online.
2. Derogatory email, text, or social media post.
3. Defaced property with derogatory graffiti.
4. Embarrassed, humiliated, or threatened by someone in person or online.

- ◯ Yes
- ◯ No
- ◯ Prefer not to say

© 2026 Sain C et al. *JAMA Network Open*.

To the extent that you are comfortable answering, do you feel you have experienced verbal, written, or online harassing behaviors due to the following identities or attributes?<br>(Mark all that apply)	☐ I experienced this, but I don't know if it was because of a particular identity or attribute<br>☐ Yes, my racial or ethnic identity<br>☐ Yes, my gender identity or gender expression<br>☐ Yes, my sexual orientation<br>☐ Yes, my transgender identity<br>☐ Yes, my ability or disability status<br>☐ Yes, my socioeconomic status or social class<br>☐ Yes, my age<br>☐ Yes, my national origin or citizenship status<br>☐ Yes, my accent or the way I speak<br>☐ I do not believe these harassing behaviors were directed at me<br>☐ Yes, an identity or attribute not listed above. (If you select this, you will be asked a follow-up question)<br>☐ Prefer not to say
If comfortable, please specify which identity or attribute which was not listed in the prior list.	
You indicated that you experienced verbal, written or online harassing behaviors.<br><br>How frequently have you experienced these harassing behaviors during the last year?	◯ Once or twice<br>◯ Once a month or less<br>◯ Two or three times a month<br>◯ Once or twice a week<br>◯ Nearly every day or every day<br>◯ Prefer not to say
At the time of these events, what was the relationship between you and the people who behaved in verbal, written, or online harassing ways towards you?<br>(Mark all that apply)	☐ Attending<br>☐ Fellow<br>☐ Resident<br>☐ Peer/Colleague (for trainees: same year training)<br>☐ Administrative staff<br>☐ Boss or supervisor<br>☐ Patient<br>☐ Nursing<br>☐ Anesthesia techs<br>☐ SRNA<br>☐ CRNA<br>☐ Prefer not to say
You indicated that you experienced verbal, written or online harassing behaviors on the basis of your gender identity, gender expression, or sexual orientation.<br><br>Have you experienced any of the following forms of verbal, written, or online sexually harassing behaviors during the last year:<br><br>Someone in your work environment ...	☐ Made a sexist remark<br>☐ Mistreated, slighted, insulted, or was condescending to you, or ignored you because of your gender<br>☐ Made inappropriate or offensive comments about your or someone else's body, appearance, or sexual activities<br>☐ Continued to ask you to go out, get dinner, have drinks, or have sex even though you said "No"<br>☐ I experienced a different form of gender-based or sexually harassing behaviors not listed above.<br>☐ No, I did not experience any of these sexually harassing behaviors<br>☐ Prefer not to say

© 2026 Sain C et al. *JAMA Network Open*.

Did these verbal, written or online harassing behaviors affect you in any of the following ways? (Mark all that apply)

- ☐ Interfered with your academic or professional performance
- ☐ Limited your ability or participate in an academic or workplace program
- ☐ Created an intimidating, hostile, or offensive social, academic, or work environment
- ☐ I was impacted, but not in these ways
- ☐ I was not significantly impacted by these experiences
- ☐ Prefer not to say

Physical Harassing Behaviors

REGARDING FORMAL REPORTING MECHANISMS

Please note, survey feedback is anonymous and confidential. While we encourage candid feedback, this survey should not be used as a reporting mechanism and no one from the university or hospital will be able to follow-up with you directly based on your responses. Survey responses will be anonymized and presented as themes.

We are committed to creating an inclusive workplace that is free of discrimination, bias and harassment where all individuals feel respected and safe. If you have a concern about your workplace experience, please let us know. Reporting options include notifying your supervisor, any manager, MGB Compliance or Human Resources via the 'Report a Concern' tile on Vitals.

During the last year, have you experienced physically harassing behaviors?

- ◯ Yes
- ◯ No
- ◯ Prefer not to say

Physically harassing behaviors examples:

1. I was threatened with physical violence.
2. I experienced physical violence.
3. Someone tried to touch me without my consent.
4. I was touched in a way that I did not want.

© 2026 Sain C et al. *JAMA Network Open*.

You indicated that you experienced physical harassing behaviors.<br><br>To the extent that you are comfortable answering, do you feel you have experienced physical harassing behaviors due to the following identities or attributes?<br><br>(Mark all that apply)	☐ I experienced this, but I don't know if it was because of a particular identity or attribute<br>☐ Yes, my racial or ethnic identity<br>☐ Yes, my gender identity or gender expression<br>☐ Yes, my sexual orientation<br>☐ Yes, my transgender identity<br>☐ Yes, my ability or disability status<br>☐ Yes, my socioeconomic status or social class<br>☐ Yes, my age<br>☐ Yes, my national origin or citizenship status<br>☐ Yes, my accent or the way I speak<br>☐ I do not believe these harassing behaviors were directed at me<br>☐ Yes, an identity or attribute not listed above. (If you select this, you will be asked a follow-up question)<br>☐ Prefer not to say
If comfortable, please specify which identity or attribute which was not listed in the prior list.	
You indicated that you experienced physical harassing behaviors.<br><br>How frequently have you experienced physical harassing behaviors during the last year?	◯ Once or twice<br>◯ Once a month or less<br>◯ Two or three times a month<br>◯ Once or twice a week<br>◯ Nearly every day or every day<br>◯ Prefer not to say
At the time of these events, what was the relationship between you and the people who behaved in physically harassing ways towards you?<br>(Mark all that apply)	☐ Attending<br>☐ Fellow<br>☐ Resident<br>☐ Peer/Colleague (for trainees: same year training)<br>☐ Administrative staff<br>☐ Boss or supervisor<br>☐ Patient<br>☐ Nursing<br>☐ Anesthesia techs<br>☐ SRNA<br>☐ CRNA<br>☐ Prefer not to say
Did these physical harassing behaviors affect you in any of the following ways?<br>(Mark all that apply)	☐ Interfered with your academic or professional performance<br>☐ Limited your ability or participate in an academic or workplace program<br>☐ Created an intimidating, hostile, or offensive social, academic, or work environment<br>☐ I was impacted, but not in these ways<br>☐ I was not significantly impacted by these experiences<br>☐ Prefer not to say

© 2026 Sain C et al. *JAMA Network Open*.

Discriminatory Behavior

REGARDING FORMAL REPORTING MECHANISMS

Please note, survey feedback is anonymous and confidential. While we encourage candid feedback, this survey should not be used as a reporting mechanism and no one from the university or hospital will be able to follow-up with you directly based on your responses. Survey responses will be anonymized and presented as themes.

We are committed to creating an inclusive workplace that is free of discrimination, bias and harassment where all individuals feel respected and safe. If you have a concern about your workplace experience, please let us know. Reporting options include notifying your supervisor, any manager, MGB Compliance or Human Resources via the 'Report a Concern' tile on Vitals.

During the last year, have you experienced discriminatory behavior?

- ◯ Yes
- ◯ No
- ◯ Prefer not to say

Examples for trainees:

Unfair evaluation, discouraged from participation in a research opportunity or a program, discouraged or denied from participation in a social event, denied or overlooked for mentorship opportunities, denied necessary accommodations.

Examples for faculty/clinicians:

Not appropriately considered for promotion, denied or overlooked for leadership positions.

Examples for administrative staff:

Denied or overlooked for a promotion, unfair or unjust hiring practice, unfair or unjust disciplinary action, denied equitable compensation.

© 2026 Sain C et al. *JAMA Network Open*.

You indicated that you experienced discriminatory behaviors.<br><br>Do you think that any of the following reasons may have contributed?	☐ I experienced this, but I don't know if it was because of a particular identity or attribute<br>☐ Yes, my racial or ethnic identity<br>☐ Yes, my gender identity or gender expression<br>☐ Yes, my sexual orientation<br>☐ Yes, my transgender identity<br>☐ Yes, my ability or disability status<br>☐ Yes, my socioeconomic status or social class<br>☐ Yes, my age<br>☐ Yes, my national origin or citizenship status<br>☐ Yes, my accent or the way I speak<br>☐ I do not believe these harassing behaviors were directed at me<br>☐ Yes, an identity or attribute not listed above. (If you select this, you will be asked a follow-up question)<br>☐ Prefer not to say
If comfortable, please specify which identity or attribute which was not listed in the prior list.	
You indicated that you experienced discriminatory behaviors.<br><br>How frequently have you experienced discrimination during the last year?	○ Once or twice<br>○ Once a month or less<br>○ Two or three times a month<br>○ Once or twice a week<br>○ Nearly every day or every day<br>○ Prefer not to say
At the time of these events, what was the relationship between you and the people who behaved in discriminatory ways towards you?<br>(Mark all that apply)	☐ Attending<br>☐ Fellow<br>☐ Resident<br>☐ Peer/Colleague (for trainees: same year training)<br>☐ Administrative staff<br>☐ Boss or supervisor<br>☐ Patient<br>☐ Nursing<br>☐ Anesthesia techs<br>☐ SRNA<br>☐ CRNA<br>☐ Prefer not to say
Did these discriminatory behaviors affect you in any of the following ways?<br>(Mark all that apply)	☐ Interfered with your academic or professional performance<br>☐ Limited your ability or participate in an academic or workplace program<br>☐ Created an intimidating, hostile, or offensive social, academic, or work environment<br>☐ I was impacted, but not in these ways<br>☐ I was not significantly impacted by these experiences<br>☐ Prefer not to say

© 2026 Sain C et al. *JAMA Network Open*.

Microaggressions

REGARDING FORMAL REPORTING MECHANISMS

Please note, survey feedback is anonymous and confidential. While we encourage candid feedback, this survey should not be used as a reporting mechanism and no one from the university or hospital will be able to follow-up with you directly based on your responses. Survey responses will be anonymized and presented as themes.

We are committed to creating an inclusive workplace that is free of discrimination, bias and harassment where all individuals feel respected and safe. If you have a concern about your workplace experience, please let us know. Reporting options include notifying your supervisor, any manager, MGB Compliance or Human Resources via the 'Report a Concern' tile on Vitals.

During the last year, has someone invalidated your individual experience due to your racial and ethnic identity?

Examples:

Someone told me that they "don't see color" or we should not think about race anymore.
Someone told me that people of color do not experience racism anymore.
Others assume that people of my racial background would succeed if they simply worked harder.
Someone assumed that I had a particular skill set due to my race or ethnicity (e.g., good at math and science, athletic ability, etc.)

- ◯ Yes
- ◯ No
- ◯ Prefer not to say

During the last year, has someone assumed you were inferior?

Examples:
Someone told me that I was "articulate" after she/he/they assumed I would not be.
Someone acted surprised at my scholastic or professional success.
Someone assumed that I was poor.
Someone assumed I come from a disadvantaged background.

- ◯ Yes
- ◯ No
- ◯ Prefer not to say

© 2026 Sain C et al. *JAMA Network Open*.

Question	Response options
During the last year, has someone made you feel othered?<br><br>Examples:<br>Someone did not believe me when I told them I was born in the U.S.<br>Some assumed that I spoke a language other than English.<br>Someone told me that all people in my racial group look alike or are all the same.	◯ Yes<br>◯ No<br>◯ Prefer not to say
You indicated that you experienced microaggressive behaviors.<br><br>How many times did someone behave in these ways towards you during the last year?	◯ Once or twice<br>◯ Once a month or less<br>◯ 2-3 times a month<br>◯ Once or twice a week<br>◯ Nearly every day or every day<br>◯ Prefer not to say
How pervasively have you experienced microaggressive behaviors during the last year?<br>(Mark all that apply)	☐ By one or two people<br>☐ By several people that you interact with<br>☐ By various groups of people in our department<br>☐ Generally institution wide<br>☐ Prefer not to say
At the time of these events, what was the relationship between you and the people who microaggressed you?<br>(Mark all that apply)	☐ Attending<br>☐ Fellow<br>☐ Resident<br>☐ Peer/Colleague (for trainees: same year training)<br>☐ Administrative staff<br>☐ Boss or supervisor<br>☐ Patient<br>☐ Nursing<br>☐ Anesthesia techs<br>☐ SRNA<br>☐ CRNA<br>☐ Prefer not to say
Did these microaggressive behaviors affect you in any of the following ways?<br>(Mark all that apply)	☐ Interfered with your academic or professional performance<br>☐ Limited your ability or participate in an academic or workplace program<br>☐ Created an intimidating, hostile, or offensive social, academic, or work environment<br>☐ I was impacted, but not in these ways<br>☐ I was not significantly impacted by these experiences<br>☐ Prefer not to say

© 2026 Sain C et al. *JAMA Network Open*.

Final Thoughts

REGARDING FORMAL REPORTING MECHANISMS

© 2026 Sain C et al. *JAMA Network Open*.

Please note, survey feedback is anonymous and confidential. While we encourage candid feedback, this survey should not be used as a reporting mechanism and no one from the university or hospital will be able to follow-up with you directly based on your responses. Survey responses will be anonymized and presented as themes.

We are committed to creating an inclusive workplace that is free of discrimination, bias and harassment where all individuals feel respected and safe. If you have a concern about your workplace experience, please let us know. Reporting options include notifying your supervisor, any manager, MGB Compliance or Human Resources via the 'Report a Concern' tile on Vitals.

© 2026 Sain C et al. *JAMA Network Open*.

If you indicated you have had: Experience with harassing behaviors, Experience with discriminatory behaviors, and/or negative intrapersonal experiences; please consider the question below.

Reflecting on the types of experiences listed above, we would like to ask about the impact these experiences have had on you.

Did these experiences affect you in any of the following ways?

(Mark all that apply)

- ☐ Avoided departmental or professional events (social)
- ☐ Avoided departmental or professional events (educational)
- ☐ Changed my daily routine to avoid certain people or places
- ☐ Changed my research interest
- ☐ Had difficulty concentrating on clinical or work-related responsibilities
- ☐ Rarely feel comfortable voicing my opinion
- ☐ Changed my academic path or clinical focus (subspecialty or fellowship interest)
- ☐ Felt ostracized or excluded
- ☐ Felt nervous, anxious, or on edge
- ☐ Not being able to stop or control worrying
- ☐ Trouble relaxing
- ☐ Being restless that it was hard to sit still
- ☐ Becoming easily annoyed or irritable
- ☐ Feeling afraid as if something awful might happen
- ☐ Had to withdraw from or request a change in rotation or assignment
- ☐ Withdrew from interactions with friends or family
- ☐ Took a temporary leave of absence
- ☐ Considered taking a leave of absence
- ☐ Decreased participation or interest in extra-professional activities, personal interests, or hobbies
- ☐ Increased feelings of down, depression, or hopelessness
- ☐ Poor appetite or overeating
- ☐ Increased fatigue or having little energy
- ☐ Feeling bad about yourself or that you are a failure or have let down you or your family
- ☐ Seriously considered leaving the program, department, or institution
- ☐ Experienced trouble falling asleep or sleeping too much
- ☐ Had difficulty attending meetings
- ☐ Increased drug or alcohol use
- ☐ Had difficulty concentrating on non-work-related tasks
- ☐ Having thoughts that you would be better off dead or hurting yourself in some other way
- ☐ Moving or speaking so slowly that other people could have noticed-- or the opposite and being so fidgety or restless that you have been moving around a lot more than usual.
- ☐ Changed office or supervisor
- ☐ I was not seriously impacted by these experiences
- ☐ I was impacted, but not in the ways listed above (If you feel comfortable, please specify on the next page)
- ☐ Prefer not to say

I was impacted, but not in the ways on the previous page. If you feel comfortable doing so, please specify here.

© 2026 Sain C et al. *JAMA Network Open*.

You selected that you experienced one or more of the negative behaviors previously mentioned.<br>Please answer the following question:<br><br>Did you make a formal report to the Department or Institution about these experiences?<br><br>For example:<br><br>Did you formally report to the Department Chief or Chair, Manager/Supervisor, Human Resources, Colleague, or Program Director?	◯ Yes<br>◯ No<br>◯ Prefer not to say
Why did you decide not to make a formal report? (Mark all that apply)	☐ I did not know where to go or who to tell<br>☐ I felt embarrassed, ashamed, or that it would be too emotionally difficult<br>☐ I did not think anyone would believe me<br>☐ I did not think the person or program I would report to would take it seriously<br>☐ I did not think it was serious to make a formal report<br>☐ I did not want the person to get into trouble<br>☐ I feared negative social consequences<br>☐ I feared negative professional consequences<br>☐ I feared it would not be kept confidential<br>☐ I feared being labeled as difficult or a complainer<br>☐ I could handle it myself<br>☐ I I feared retaliation<br>☐ I did not think a formal report would give me the help I needed<br>☐ Incident outside of a professional or clinical setting.<br>☐ The reason is not listed above<br>☐ Prefer not to say
Did you contact any other person or resources for advice, support, or information?	◯ Yes<br>◯ No<br>◯ Not Applicable<br>◯ Prefer not to say

Thank you for taking the time to fill out this survey. You can click the attached document for Peer Support Resources if you would like to see what resources are available.

At this time you may click submit as it is now complete.

REGARDING FORMAL REPORTING MECHANISMS

Please note, survey feedback is anonymous and confidential. While we encourage candid feedback, this survey should not be used as a reporting mechanism and no one from the university or hospital will be able to follow-up with you directly based on your responses. Survey responses will be anonymized and presented as themes.

We are committed to creating an inclusive workplace that is free of discrimination, bias and harassment where all individuals feel respected and safe. If you have a concern about your workplace experience, please let us know. Reporting options include notifying your supervisor, any manager, MGB Compliance or Human Resources via the 'Report a Concern' tile on Vitals.

[Attachment: "Support Resources.pdf"]

© 2026 Sain C et al. *JAMA Network Open*.
